# Pre‐Fracture Cognitive Assessment Using the DASC‐21 and Postoperative Delirium Risk

**DOI:** 10.1111/psyg.70195

**Published:** 2026-07-24

**Authors:** Tomoko Kamimura, Satoshi Tamaki, Yuya Kobayashi, Keita Tomii

**Affiliations:** ^1^ Department of Health Sciences Shinshu University School of Medicine Matsumoto Nagano Japan; ^2^ Kamiiida‐Daiichi General Hospital Nagoya Aichi Japan; ^3^ Aizawa Hospital Matsumoto Nagano Japan

**Keywords:** cognitive impairment, delirium, dementia, hip fracture, risk factor

## Abstract

**Background:**

Postoperative delirium (POD) is a common complication in older adults after hip fracture surgery. Although cognitive impairment is a key risk factor, accurately assessing baseline cognition in acute settings remains challenging. Informant‐based tools may better reflect premorbid cognitive status; however, their utility in hip fracture populations is unclear.

**Methods:**

This retrospective analysis used data from a prospective cohort of patients aged ≥ 80 years who underwent hip fracture surgery at two acute hospitals in Japan. Pre‐fracture cognitive status was assessed using the Dementia Assessment Sheet for Community‐based Integrated Care System (DASC‐21), primarily based on reports from informants. For selected patients without available informants and with Mini‐Mental State Examination (MMSE) scores ≥ 21, structured patient interviews and clinical observations were used. The DASC‐21 categories were classified as normal cognition, mild impairment and moderate‐to‐severe impairment. Cognitive function at admission was evaluated using the MMSE. POD was assessed using the Confusion Assessment Method within the first three postoperative days. Multivariable logistic regression analyses were performed.

**Results:**

Among 368 patients, 137 (37.2%) developed POD. POD incidence increased with worsening pre‐fracture cognitive impairment (5.6%, 44.3% and 48.7% for normal, mild and moderate‐to‐severe impairment, respectively; *p* < 0.001). Each 1‐point increase in the MMSE score was associated with lower odds of POD (OR 0.91, 95% CI 0.88–0.94). Compared with normal cognition, the adjusted ORs for POD were 14.11 (95% CI 5.08–39.24) for mild impairment and 15.39 (95% CI 5.56–42.61) for moderate‐to‐severe impairment. Discrimination was similar between MMSE and DASC‐21 (area under the receiver operating characteristic curve 0.75 vs. 0.74).

**Conclusions:**

Pre‐fracture cognitive impairment, including DASC‐21‐defined mild impairment, was associated with POD. The DASC‐21 categories showed comparable discriminative performance to the MMSE and may help estimate baseline cognitive vulnerability in acute clinical settings.

## 1 Introduction

Hip fractures are a major health concern among older adults, particularly those aged ≥ 80 years [1]. Postoperative delirium (POD) is a common complication after hip fracture surgery [2, 3, 4, 5], and is associated with prolonged hospitalization [6], functional decline [7] and increased mortality [2, 5]. Therefore, the early identification of high‐risk patients is essential [8, 9].

Cognitive impairment is a well‐established risk factor for POD development. While prior studies have primarily focused on overt or moderate‐to‐severe impairment [4, 10, 11, 12, 13, 14, 15, 16, 17, 18], the impact of mild impairment in hip fracture populations remains unknown. Evidence from elective surgery suggests that even mild impairment increases the risk of delirium [19, 20]; however, its role in urgent surgical settings is less well understood.

A key challenge in this context is the accurate assessment of the baseline cognitive status. Cognitive testing at admission may be influenced by acute factors such as pain, stress, or early delirium, potentially leading to misclassification of pre‐existing impairments. Therefore, approaches that better reflect the premorbid cognitive status are needed.

Informant‐based assessments may address this limitation by capturing habitual cognitive and functional decline. The Dementia Assessment Sheet for Community‐based Integrated Care System (DASC‐21) is a validated instrument designed to assess cognitive and functional status based on informant reports [21, 22]. The DASC‐21 evaluates memory, orientation, problem solving, instrumental ADL and physical ADL, and may help identify early cognitive and functional decline and dementia risk in community‐dwelling older adults. However, its utility in acute hip fracture settings has not been well established.

This study aimed to examine the association between pre‐fracture cognitive impairment, including DASC‐21‐defined mild impairment, and POD in older adults with hip fractures and to evaluate the clinical utility of informant‐based cognitive assessment using the DASC‐21.

## 2 Methods

### 2.1 Study Design and Participants

This retrospective analysis used data from a prospective cohort study [23] conducted at two acute hospitals in Japan. Patients aged ≥ 80 years who underwent surgery for hip fractures between February (at Kamiiida‐Daiichi General Hospital) or August (at Aizawa Hospital) 2021 and July 2022 were consecutively enrolled.

Patients were excluded if major in‐hospital events occurred (e.g., death, severe illness, or additional hip fracture), if reliable assessment of pre‐fracture cognitive status was not possible (i.e., absence of informants in patients with Mini‐Mental State Examination (MMSE) scores < 21 [24]), or if informed consent for participation in the prospective cohort study was not obtained.

The Shinshu University Ethics Committee approved the study protocol (approval number: 6132).

### 2.2 Outcome

POD was assessed daily using the Confusion Assessment Method (CAM). Patients who met the CAM criteria at least once within the first three postoperative days were classified as having POD. This time window was selected to capture early POD, which is the most clinically relevant in hip fracture care, as delirium typically occurs within the first few days after surgery in this population.

### 2.3 Cognitive Assessment

Cognitive function at admission was assessed using the MMSE. Pre‐fracture cognitive status was evaluated using the DASC‐21, primarily through informant interviews with family members or caregivers. Participants were classified into three categories: normal cognition (DASC‐21 < 31), mild impairment and moderate‐to‐severe impairment. For participants with DASC‐21 scores ≥ 31, severity ratings across four domains—remote memory, spatial orientation, social common sense and physical ADL—were used to determine dementia risk (low, medium, or serious) [21, 22]. Those with low risk were categorised as having mild impairment, whereas those with medium or serious risk were combined into the moderate‐to‐severe‐risk group. When informants were unavailable, DASC‐21 assessments were conducted via structured patient interviews and clinical observations; this alternative approach was restricted to patients with MMSE scores ≥ 21 to minimise measurement variability.

### 2.4 Covariates

Covariates were selected based on the literature and included age [4, 10, 11, 13], sex [4, 11], place of residence [10], pre‐fracture functional dependence [4, 11] (modified Barthel Index ambulation score < 12 [25, 26]), body mass index (BMI) [11], comorbidities [4] (Charlson Comorbidity Index [CCI] ≥ 3 [27]), American Society of Anesthesiologists physical status ≥ 3 [4] and surgical delay > 2 days [4].

### 2.5 Statistical Analysis

Continuous variables are presented as medians with interquartile ranges, and categorical variables are expressed as counts and percentages. Continuous variables were compared using the Mann–Whitney *U* or Kruskal–Wallis tests. Categorical variables were compared using the chi‐square test.

Multivariable logistic regression analyses were conducted to examine the association between cognitive measures and POD. Model 1 included the MMSE score as a continuous variable, and Model 2 included the DASC‐21 categories (normal cognition, mild impairment and moderate‐to‐severe impairment). All models were adjusted for age, sex, place of residence, functional dependence, BMI, comorbidities, ASA physical status and surgical delay. Adjusted odds ratios (ORs) with 95% confidence intervals (CIs) were reported for the analysis.

To justify this two‐model structure, we evaluated a combined model that included both the MMSE and DASC‐21. If substantial multicollinearity was indicated between the two measures, we constructed two separate final models to ensure statistical validity. The results of this combined model and multicollinearity diagnostics are provided in the Supporting Information.

Model discrimination was evaluated using the area under the receiver operating characteristic curve (AUC), and the AUCs were compared using the DeLong test. The model fit was assessed using the Akaike information criterion (AIC) and Bayesian information criterion (BIC).

To confirm the robustness of our primary findings, we conducted two sensitivity analyses: (1) we categorised the MMSE scores into three levels to match the operationalization of the DASC‐21, and (2) we excluded cases where the DASC‐21 was administered directly to the patients rather than to their informants, ensuring the consistency of the informant‐based data. The results of these sensitivity analyses are presented in the Supporting Information.

A two‐sided *p* < 0.05 was considered statistically significant. All statistical analyses were performed using SPSS version 31 (IBM Corp., Armonk, NY, USA).

## 3 Results

### 3.1 Participant Characteristics

After exclusions, 368 patients were included in the analysis. Participants were categorised as having normal cognition (*n* = 89), mild impairment (*n* = 88), or moderate‐to‐severe impairment (*n* = 191) according to the DASC‐21. Exclusions included inability to complete the DASC‐21 (*n* = 2), in‐hospital death (*n* = 7), serious illness (*n* = 5), additional hip fracture (*n* = 2) and lack of consent through the opt‐out procedure (*n* = 45).

DASC‐21 assessments were based on informant interviews in 319 patients (86.7%) and structured patient interviews/clinical observations in 49 patients (13.3%).

POD occurred in 137 patients (37.2% of the total). Patients with POD were older, had lower MMSE scores and were more likely to have pre‐fracture functional dependence (Table 1).

**TABLE 1:** Baseline characteristics of the participants.

Characteristics	All (*n* = 368)	No delirium (*n* = 231)	Delirium (*n* = 137)	*p*
Age (years)	88 [7.0]	88 [8.0]	89 [7.0]	0.03
Female sex	313 (85.1%)	188 (81.4%)	125 (91.2%)	0.01
MMSE score	19 [13]	22 [12]	15 [11]	< 0.001
DASC‐21 normal cognition	89 (24.2%)	84 (36.4%)	5 (3.6%)	< 0.001
Mild impairment	88 (23.9%)	49 (21.2%)	39 (28.5%)	
Moderate‐to‐severe impairment	191 (51.9%)	98 (42.4%)	93 (67.9%)	
Place of residence, home	229 (62.2%)	159 (68.8%)	70 (51.1%)	< 0.001
Pre‐fracture functional dependence	72 (19.6%)	35 (15.2%)	37 (27.0%)	0.006
BMI (kg/m^2^)	19.8 [4.1]	19.7 [4.3]	19.9 [3.9]	0.44
CCI ≤ 2	325 (88.3%)	204 (88.3%)	121 (88.3%)	1.00
ASA class				0.29
1–2	262 (71.2%)	160 (69.3%)	102 (74.5%)	
3–4	106 (28.8%)	71 (30.7%)	35 (25.5%)	
Fracture type, intra‐articular	167 (45.4%)	106 (45.9%)	61 (44.5%)	0.80
Surgery BHA	132 (35.9%)	89 (38.5%)	43 (31.4%)	0.06
Intramedullary nail	197 (53.5%)	123 (53.3%)	74 (54.0%)	
Others	39 (10.6%)	19 (8.2%)	20 (14.6%)	
Surgical delay of ≤ 2 days	181 (49.2%)	111 (48.1%)	70 (51.1%)	0.57

*Note:* Data are presented as numbers (percentages) or medians [interquartile ranges].

Abbreviations: ASA, American Society of Anesthesiologists; BHA, bipolar hip arthroplasty; BMI, body mass index; CCI, Charlson Comorbidity Index; DASC‐21, 21‐item Dementia Assessment Sheet for the Community‐Based Care System; MMSE, Mini‐Mental State Examination.

### 3.2 Incidence of POD

**FIGURE 1:**
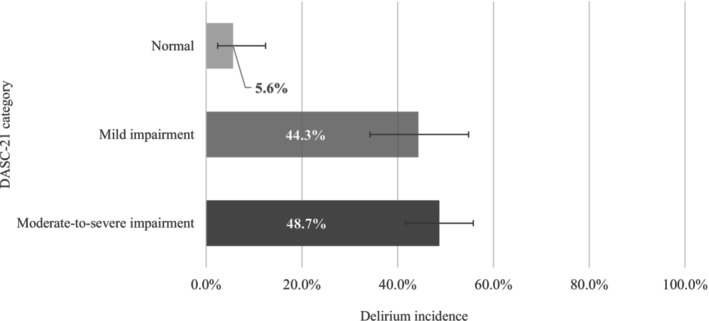
Incidence of postoperative delirium according to pre‐fracture DASC‐21 category. The incidence of postoperative delirium increased with worsening pre‐fracture cognitive impairment. Delirium occurred in 5.6% (5/89), 44.3% (39/88), and 48.7% (93/191) of patients with normal cognition, mild impairment, and moderate‐to‐severe cognitive impairment, respectively. Error bars represent 95% confidence intervals. Differences among the groups were statistically significant (*p* < 0.001).

The incidence of POD increased with worsening pre‐fracture cognitive impairment (5.6%, 44.3% and 48.7%) for normal, mild and moderate‐to‐severe impairment, respectively (*p* < 0.001; Figure 1).

Similar trends were observed when the participants were stratified according to the MMSE categories (Table S1). The incidence of POD also increased with worsening DASC‐21 category among informant‐only cases (Table S2).

### 3.3 Multivariable Analysis

Each 1‐point increase in the MMSE score was associated with a 9% lower odds of POD (OR 0.91, 95% CI 0.88–0.94). The DASC‐21 categories were also associated with POD, with adjusted ORs of 14.11 for mild impairment and 15.39 for moderate‐to‐severe impairment compared with normal cognition (Table 2).

**TABLE 2:** Multivariate analysis of factors influencing the onset of delirium.

Variables	Model 1: MMSE OR (95% CI)	*p*	Model 2: DASC OR (95% CI)	*p*
MMSE (per 1‐point increase)	0.91 (0.88–0.94)	< 0.001	—	—
DASC‐21				
Mild impairment (vs. normal)	—	—	14.11 (5.08–39.24)	< 0.001
Moderate‐to‐severe impairment (vs. normal)	—	—	15.39 (5.56–42.61)	< 0.001
Age (per 1‐year increase)	1.02 (0.97–1.07)	0.53	1.00 (0.95–1.05)	0.93
Male (vs. female)	0.55 (0.27–1.16)	0.12	0.48 (0.23–1.01)	0.05
Institutional residence (vs. home)	0.94 (0.55–1.61)	0.82	0.99 (0.59–1.66)	0.96
Pre‐fracture functional dependence (vs. independence)	1.24 (0.68–2.26)	0.49	1.24 (0.69–2.24)	0.47
BMI (per kg/m^2^)	1.00 (0.93–1.08)	0.95	0.97 (0.91–1.05)	0.48
CCI ≥ 3 (vs. 0–2)	1.13 (0.51–2.48)	0.77	1.10 (0.50–2.41)	0.82
ASA class 3–4 (vs. 1–2)	0.78 (0.44–1.36)	0.38	0.66 (0.38–1.15)	0.14
Surgical delay > 2 days (vs. 0–2 days)	0.95 (0.59–1.52)	0.81	0.86 (0.53–1.40)	0.56
	AIC = 445.08 BIC = 484.13 AUC = 0.75		AIC = 436.97 BIC = 479.93 AUC = 0.74	

*Note:* Model 1 includes MMSE. Model 2 includes the DASC‐21 categories. Both models were adjusted for age, sex, residence, functional dependence, BMI, comorbidities, ASA class and surgical delay.

Abbreviations: AIC, Akaike information criterion; ASA, American Society of Anesthesiologists; AUC, area under the receiver operating characteristic curve; BIC, Bayesian information criterion; BMI, body mass index; CCI, Charlson Comorbidity Index; DASC‐21, 21‐item Dementia Assessment Sheet for the Community‐based Care System; MMSE, Mini‐Mental State Examination.

Model discrimination was similar between the MMSE and DASC‐21 models (AUC 0.75 vs. 0.74), and there was no significant difference in the AUC between the two models according to the DeLong test (*p* = 0.689).

A multivariable logistic regression model, including both MMSE and DASC‐21, was constructed to examine their combined association with POD (Table S3). Model discrimination showed only minimal improvement in the combined model (AUC 0.77). MMSE and DASC‐21 scores were strongly correlated (Spearman's *ρ* = −0.713), and the variance inflation factors suggested multicollinearity.

Sensitivity analyses restricted to cases with informant‐based DASC‐21 assessments showed similar trends (Table S4).

## 4 Discussion

In this study, pre‐fracture cognitive impairment assessed using the DASC‐21 was independently associated with POD in older adults who underwent hip fracture surgery. Notably, even DASC‐21‐defined mild impairment was associated with an increased risk of POD development. Because the DASC‐21 incorporates both cognitive and functional domains, the mild impairment category should not be interpreted as being equivalent to clinically defined mild cognitive impairment (MCI). However, it may reflect an intermediate stage of cognitive and functional decline before dementia. Sensitivity analyses restricted to cases with informant‐based DASC‐21 assessments showed similar overall trends.

These findings are consistent with previous reports suggesting that clinically mild cognitive dysfunction is associated with an increased risk of POD in patients undergoing elective surgery [19, 20]. Although the estimated risks for mild and moderate‐to‐severe impairment were of similar magnitudes, these differences should be interpreted cautiously, given the overlap in the CIs. Rather than indicating a clear severity‐dependent relationship, the findings suggest that even DASC‐21‐defined mild impairments may increase the vulnerability to delirium.

An important implication of this study is the potential role of informant‐based cognitive assessments in acute care settings. Although the MMSE and DASC‐21 showed similar discrimination, they assessed different constructs. The MMSE reflects cognitive performance at admission and may be influenced by acute factors, such as pain, stress, or early delirium. In contrast, the DASC‐21 was designed to capture premorbid cognitive and functional status based on informant reports. Therefore, it may provide a complementary perspective on baseline cognitive vulnerability, particularly in situations where direct cognitive testing is unreliable or not feasible. However, because a minority subset of patients without available informants underwent DASC‐21 assessment using structured patient interviews and clinical observations, the findings should not be interpreted as representing a purely informant‐based assessment approach.

To further explore this relationship, a combined model including both the MMSE and DASC‐21 was examined as a secondary analysis. However, owing to multicollinearity between the two measures and only marginal improvement in discriminatory performance, the combined model resulted in attenuation and instability of the effect estimates. Therefore, these results are presented in Table S3.

The magnitude of the observed associations in the primary model should be interpreted cautiously. Because the incidence of POD in the normal cognition group was very low (5.6%), the resulting odds ratios may appear disproportionately large because of the mathematical properties of logistic regression, despite more modest absolute differences between groups. In addition, the overlapping CIs suggest that the differences between mild and moderate‐to‐severe impairment should not be interpreted as evidence of a clear dose–response relationship. Furthermore, the decrease in odds ratios in the sensitivity analysis (Table S4) compared to the primary analysis suggests that the inclusion of patients evaluated via patient interviews (who had relatively preserved cognition and a lower baseline risk of POD) may have influenced the magnitude of the estimated odds ratios in the overall model. However, we retained the overall cohort as the primary analysis to reflect the real‐world clinical setting, where informants are not always available.

Although the discriminatory performances of the MMSE and DASC‐21 models were comparable, neither achieved high predictive accuracy (AUC approximately 0.75). This suggests that cognitive measures alone may not be sufficient for precise risk prediction and that additional clinical or perioperative factors need to be considered.

Additionally, several limitations should be considered. First, in cases without available informants, DASC‐21 assessments were estimated using structured patient interviews and clinical observations. Although this approach was limited to patients with relatively preserved cognition (MMSE ≥ 21), some measurement variability and information bias cannot be excluded. Furthermore, excluding patients without informants and those with severe cognitive impairment may have limited generalizability. Second, although the CCI was included in the adjusted analyses, older adults with hip fracture often present with complex multimorbidity and frailty that may not be fully captured by the CCI alone. Medication‐related risk factors for delirium, such as pre‐ or postoperative anticholinergic or benzodiazepine use, were also unmeasured and residual confounding due to these unmeasured factors may remain. Third, approximately 10.9% of eligible patients opted out of the study, raising the possibility of selection bias because detailed data on non‐participants were unavailable. Finally, as the study was conducted at two hospitals in Japan, the findings may not be fully generalizable to other acute care settings.

In conclusion, prefracture cognitive impairment, including DASC‐21‐defined mild impairment, was significantly associated with POD. The DASC‐21 assessment may complement performance‐based measures such as the MMSE by providing additional clinical insight into the baseline cognitive status in acute orthogeriatric settings.

## Funding

This work was supported by JSPS KAKENHI (grant numbers JP19k11320 and JP23k10399).

## Ethics Statement

The research protocol was approved by the Shinshu University Ethics Committee in accordance with the Declaration of Helsinki (reference number: 6132). An opt‐out procedure approved by the ethics committees was implemented, and explanatory documents were posted in the facility to allow participants to decline participation if they wished.

## Conflicts of Interest

The authors declare no conflicts of interest.

## Supporting information


**Table S1:** Incidence of postoperative delirium according to MMSE category (*n* = 368).
**Table S2:** Incidence of postoperative delirium according to the DASC‐21 category: Informant‐based Cases Only (*n* = 319).
**Table S3:** Multivariable logistic regression model including both MMSE and DASC‐21.
**Table S4:** Multivariate analysis of factors influencing the onset of delirium: Cases Involving Only Informants (*n* = 319).

## Data Availability

The data that support the findings of this study are available on request from the corresponding author. The data are not publicly available due to privacy or ethical restrictions.
