# Supplementary material for: Pre‐Fracture Cognitive Assessment Using the DASC‐21 and Postoperative Delirium Risk
Source: Psychogeriatrics. 2026 Jul 24;26(5):e70195. doi: 10.1111/psyg.70195 (PMC13396967; doi:10.1111/psyg.70195)
Supplement: Supplementary file 1 — Table S1: Incidence of postoperative delirium according to MMSE category (n = 368). Table S2: Incidence of postoperative delirium according to the DASC‐21 category: Informant‐based Cases Only (n = 319). Table S3: Multivariable logistic regression model including both MMSE and DASC‐21. Table S4: Multivariate analysis of factors influencing the onset of delirium: Cases Involving Only Informants (n = 319). [file PSYG-26-0-s001.docx]

Supplementary Material

Table S1 Incidence of postoperative delirium according to MMSE category (n=368)

| MMSE category | Delirium Incidence |
| --- | --- |
| Normal cognition (MMSE 24-30) (n=115) | 10 (8.7%) |
| Mild impairment; (MMSE 18-23) (n=90) | 36 (40.0%) |
| Moderate-to-severe impairment; (MMSE 0-17) (n=163) | 92 (55.8%) |

Abbreviations: MMSE, Mini Mental State Examination

Table S2 Incidence of postoperative delirium according to the DASC-21 category: Informant-based Cases Only (n=319)

| DASC-21 category | Delirium Incidence |
| --- | --- |
| Normal (n=50) | 5 (10.0%) |
| Mild impairment (n=81) | 36 (44.4%) |
| Moderate-to-severe impairment (n=188) | 92 (48.9%) |

Abbreviations: DASC-21, 21-item Dementia Assessment Sheet for the Community-based Care System;

Table S3. Multivariable logistic regression model including both MMSE and DASC-21

| Variables | MMSE+DASC  OR (95% CI) | p-value |
| --- | --- | --- |
| MMSE  (per 1-point increase) | 0.93 (0.89-0.96) | <0.001 |
| DASC-21 |  |  |
| Mild impairment  (vs. normal) | 10.14 (3.60-28.56) | <0.001 |
| Moderate-to-severe impairment (vs. normal) | 6.42 (2.13-19.38) | <0.001 |
| Age (per 1-year increase) | 1.00 (0.95-1.05) | 0.86 |
| Male (vs. female) | 0.52 (0.24-1.12) | 0.10 |
| Institutional residence  (vs. home) | 0.83 (0.48-1.43) | 0.49 |
| Pre-fracture functional dependence (vs. independence) | 1.22 (0.66-2.25) | 0.52 |
| BMI (per kg/m²) | 0.99 (0.92-1.07) | 0.78 |
| CCI ≥3 (vs. 0-2) | 1.12 (0.50-2.51) | 0.78 |
| ASA class 3-4 (vs. 1-2) | 0.71 (0.40-1.27) | 0.25 |
| Surgical delay >2 days  (vs. 0-2 days) | 0.83 (0.51-1.37) | 0.47 |
|  | AIC=422.73  BIC=469.59  AUC=0.77 | |

A multivariable logistic regression model, including both MMSE and DASC-21 categories, was constructed to explore their combined association with postoperative delirium.

MMSE and DASC-21 scores were moderately to strongly correlated (Spearman’s ρ = −0.713), and the variance inflation factors suggested the presence of multicollinearity. When both variables were included, the effect estimates were less stable than those of models including each variable separately.

Model discrimination showed only limited improvement compared with the individual models (AUC 0.77 vs. 0.75 and 0.74 for the MMSE and DASC-21 models, respectively), and improvements in model fit were modest.

Given these findings, the combined model is presented as supplementary material.

Abbreviations: MMSE, Mini-Mental State Examination; DASC-21, 21-item Dementia Assessment Sheet for the Community-based Care System; BMI, body mass index; CCI, Charlson Comorbidity Index; ASA, American Society of Anesthesiologists; AIC, Akaike information criterion; BIC, Bayesian information criterion; AUC, area under the receiver operating characteristic curve

Table S4 Multivariate analysis of factors influencing the onset of delirium: Cases Involving Only Informants (n=319)

| Variables | Model 2: DASC OR (95% CI) | p-value |
| --- | --- | --- |
| DASC-21 |  |  |
| Mild impairment  (vs. normal) | 7.60 (2.67-21.69) | <0.001 |
| Moderate-to-severe impairment (vs. normal) | 8.72 (3.09-24.56) | <0.001 |
| Age (per 1-year increase) | 1.00 (0.95-1.05) | 0.93 |
| Male (vs. female) | 0.52 (0.24-1.13) | 0.10 |
| Institutional residence  (vs. home) | 0.93 (0.55-1.58) | 0.79 |
| Pre-fracture functional dependence (vs. independence) | 1.22 (0.67-2.21) | 0.51 |
| BMI (per kg/m²) | 0.97 (0.90-1.05) | 0.48 |
| CCI ≥3 (vs. 0-2) | 1.14 (0.52-2.50) | 0.75 |
| ASA class 3-4 (vs. 1-2) | 0.58 (0.33-1.03) | 0.06 |
| Surgical delay >2 days  (vs. 0-2 days) | 0.92 (0.56-1.50) | 0.74 |
|  | AIC=417.02  BIC=459.98  AUC=0.69 | |

Abbreviations: DASC-21, 21-item Dementia Assessment Sheet for the Community-based Care System; BMI, body mass index; CCI, Charlson Comorbidity Index; ASA, American Society of Anesthesiologists; AIC, Akaike information criterion; BIC, Bayesian information criterion; AUC, area under the receiver operating characteristic curve.
